# Bilateral Supernumerary Deciduous Maxillary Lateral Incisors with Fusion: Report of a Rare Case

**Published:** 2016-03

**Authors:** Faezeh Ghaderi, Azade Rafiee

**Affiliations:** 1 Dept. of Pediatric Dentistry, School of Dentistry, Shiraz University of Medical Sciences, Shiraz, Iran.; 2 Postgraduate Student of Research Committee, Dept. of Pediatric Dentistry, School of Dentistry, Shiraz University of Medical Sciences, Shiraz, Iran.

**Keywords:** Supernumerary Tooth, Deciduous Tooth, Fusion

## Abstract

Dental anomaly in number, size and shape might be due to excessive activation of dental lamina during the morpho-differentiation stage. Fusion is one of the most unusual and rare anomalies of shape of the teeth. It frequently involves the supernumerary teeth resulting in a challenging differential diagnosis with gemination. Dental anomalies may result in different problems such as delayed eruption and crowding; thus, early diagnosis is required for effective intervention and proper in-time treatment. The case reported here is a 4-year-old girl with bilateral supernumerary primary maxillary lateral incisors associated with fusion between primary maxillary left lateral incisor and supernumerary lateral tooth.

## Introduction

Developmental anomalies during the morpho-differentiation stage of the dental lamina and the tooth germ may cause abnormalities in the number, size and shape of the teeth such as fusion, gemination, dental twinning, and concrescence.[1] Supernumerary teeth are described as an anomaly in number of teeth due to the excessive normal dental lamina activity.[2-4] Supernumerary teeth occur rarely in primary dentition compared to the permanent dentition.[5-7] The prevalence of supernumerary teeth in primary dentition is reported to be 0.2–1.9%.[8-9] Males are affected nearly twice than the females.[10]

Supplemental teeth may result in esthetic problems, delayed eruption, and crowding; thus, early diagnosis and treatment of them is requisite. Sometimes, the supernumerary tooth may be fused to the normal tooth leading to a complicated diagnosis.

The fusion occurs because of the complete or partial union of dentin or enamel of two or more developing tooth germs.[11] The prevalence of tooth fusion in the primary dentition is reported to be 0.5-2.5%.[3]

Hagman showed that when fusion involves primary lateral incisor and canine, the missing probability of the succedaneous lateral incisor and canine is 100% and 75%, respectively. That study also reported that when the fusion involves central and lateral incisors, missing permanent successors occurs just in 37.5% of the cases.[12]

The case reported here is a 4-year-old girl with bilateral supernumerary primary maxillary lateral incisors associated with fusion between the primary maxillary left lateral incisor and supernumerary lateral tooth. 

## Case Report

A 4-year-old girl was referred to Shiraz Dental School for routine dental check-up. Clinical examination revealed no dental caries; while, precise intra-oral examination showed abnormality in the number and size of teeth in the anterior region of maxilla. Supplemental lateral incisor caused no crowding in the upper anterior region. Familial history of these anomalies was negative based on her parents’ report, though; they were unaware of the presence of this abnormality.

**Figure 1:**
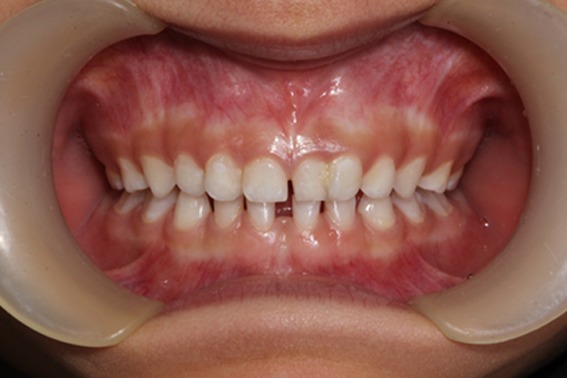
The intra-oral view. Fusion between the primary maxillary left lateral incisor and lateral supernumerary tooth is evident.

In intraoral examination, bilateral supernumerary primary maxillary lateral incisors were found (Figure 1). In the left side, fusion was found between the primary maxillary left lateral incisor and the lateral supernumerary tooth (Figure 1).

**Figure 2:**
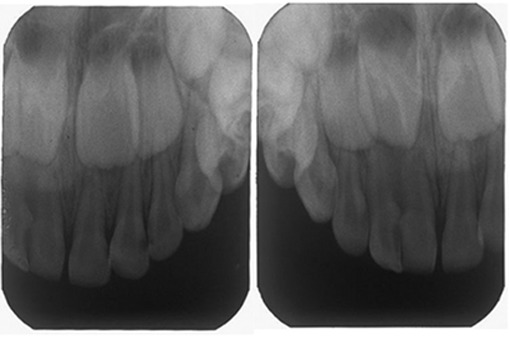
The peri-apical radiograph shows incomplete fusion of the primary maxillary left lateral incisor and the lateral supernumerary tooth. Supernumerary right lateral incisor shows a slight crown dilaceration.

In radiographic view, the fusion was partial and the involved teeth had two separated roots (Figure 2).

**Figure 3:**
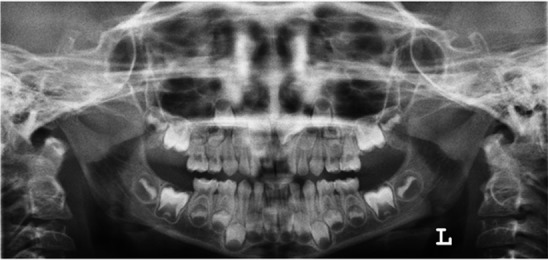
The panoramic radiograph shows no evidence for presence of supernumerary permanent tooth germ in the anterior region. However, rotation of the permanent central teeth germs and development of dens invagination in permanent maxillary right central incisor are suspected.

Panoramic and periapical radiographs revealed bilateral supplemental primary maxillary lateral incisors without the supplemental permanent maxillary teeth (Figure 2, 3).

In the radiographic view, incomplete fusion of primary maxillary left lateral incisor and the lateral supernumerary tooth was seen. Supernumerary right lateral incisor showed a slight crown dilaceration. There was no evidence of presence of supernumerary permanent tooth germ in the anterior region. However, rotation of the permanent central teeth germs and development of dens invagination in permanent maxillary right central incisor were suspected (Figure 2, 3). As her dental abnormalities had not resulted in any problem in dental health, regular follow-up was scheduled to monitor the proper time for intervention. 

## Discussion

Supernumerary teeth in primary dentition are often overlooked because they often erupt normally in normal position and shape, as it was in this case report. Regarding the fact that the supernumerary teeth usually have normal eruption and exfoliation, and many of the children do not have initial dental visit until eruption of the permanent anterior teeth, the supernumerary teeth in some cases are not detected.[13] Some of the complications that may be related to supernumerary teeth are dentigerous cyst formation, aesthetic problem, and resorption of the adjacent roots.[14]

Root resorption is extremely rare in these cases. In many cases, supernumerary teeth are not associated with any problem. They may be accidently detected in dental examination or may be found during routine radiographic evaluation. None of the patients with this anomaly showed any symptom.[5, 13]

It is noteworthy that the fusion of teeth was also found accidentally. The dental management of the fused primary teeth would be regular observation to follow up their normal exfoliation. However, in some situations, endodontic therapy, restoration, separation with restoration, or extraction can be considered if needed.[15]

Some cases were reported with the fusion of primary supernumerary teeth with the adjacent tooth. In our reported case, the child had bilateral supernumerary primary maxillary lateral incisors associated with fusion between the primary maxillary left lateral incisor and supernumerary lateral tooth, which is a rare case. Fusion in this case was partial. In the analysis of six fusion cases, More and Tailor showed partial fusion in five cases (83.3%) and complete fusion in one case (16.7%); and surprisingly only one succedaneous tooth was missing.[1]

In a case report similar to our case, Tomizawa *et al.* detected also the permanent germ of the supernumerary teeth in the radiography. The primary teeth were extracted in their reported case because they were associated with delayed eruption of permanent maxillary central incisors.[16] However, since no problem was associated with these anomalies in our case, only follow-up was scheduled.

Radiographic studies revealed that hyperdontia in permanent dentition were more observed in children with primary supernumerary teeth than the others.[17] These findings showed that our case is a very rare one since the permanent supernumerary germs of primary supplemental lateral incisors were not found even in the regions with no fusion. This finding was consistent with the results of the studies which proposed that hyperdontia is more common in males than females.[4, 18-19]

To sum up, supernumerary teeth can cause many problems in the eruption and alignment of normal teeth. Early diagnosis and treatment are, therefore, important to prevent complications. The management and treatment protocol of a supernumerary tooth should be designed as a part of complete treatment plan, not as separate problem. 
